# Dietary N-acetylcysteine enhances sperm motility by remodeling the rumen microbiome and its metabolic axis in goats

**DOI:** 10.1186/s40104-026-01390-2

**Published:** 2026-04-20

**Authors:** Jinhong Luo, Xiaodong Wang, Yonghong Ju, Quan Ji, Ruiyang Li, Yong Ruan, Jiafu Zhao, Qingmeng Long, Yishun Shang, Ping Li, Maosheng Cao, Xiang Chen

**Affiliations:** 1 College of Animal Science/Key Laboratory of Animal Genetics, Breeding and Reproduction in the Plateau Mountainous Region, Ministry of Education/Key Laboratory of Animal Genetics, Breeding and Reproduction in The Plateau Mountainous Region, Ministry of Education, Guizhou University, Guiyang, 550025 China; 2 Guizhou Institute of Prataculture, Guizhou Academy of Agricultural Science, Guiyang, China; 3 Guizhou Provincial Livestock and Poultry Germplasm Evaluation Center, Guiyang, China

**Keywords:** Goat, Multi-omics, N-acetylcysteine, Rumen microbiome, Sperm motility

## Abstract

**Background:**

Enhancing sperm motility is crucial for improving male fertility in ruminants. The rumen microbiota, central to nutrient metabolism of ruminants, represents a promising yet underexplored target for dietary intervention. This study investigated whether N-acetylcysteine (NAC) improves sperm motility via modulating the rumen microbiota–metabolite axis.

**Results:**

Dietary NAC supplementation significantly enhanced sperm motility in goats (*P* < 0.05), with the optimal effect observed at 0.3%, which coincided with improvements in sperm membrane integrity, antioxidant capacity, and mitochondrial function. Functional analysis revealed that NAC-induced microbial remodeling enriched KEGG pathways involved in antioxidant, energy, and lipid metabolism. Correspondingly, beneficial bacteria such as *Pediococcus pentosaceus*, *Bacteroides acidifaciens*, and *Akkermansia*, among others, were significantly enriched (*P* < 0.05). Notably, metabolic alterations in these pathways were consistently reflected in both the rumen fluid and plasma metabolomes, as evidenced by 25 conserved pathways and 2 overlapping metabolites. Collectively, these metabolic alterations ultimately enhanced sperm motility by improving sperm antioxidant status, energy supply, and lipid homeostasis.

**Conclusions:**

Our study thus reveals that NAC enhances sperm motility via a rumen microbiome-mediated “rumen–plasma–sperm” axis. This novel insight broadens the understanding of how NAC—and potentially other antioxidants—regulates sperm motility, highlighting the promise of NAC-based dietary interventions for improving reproductive performance.

**Graphical Abstract:**

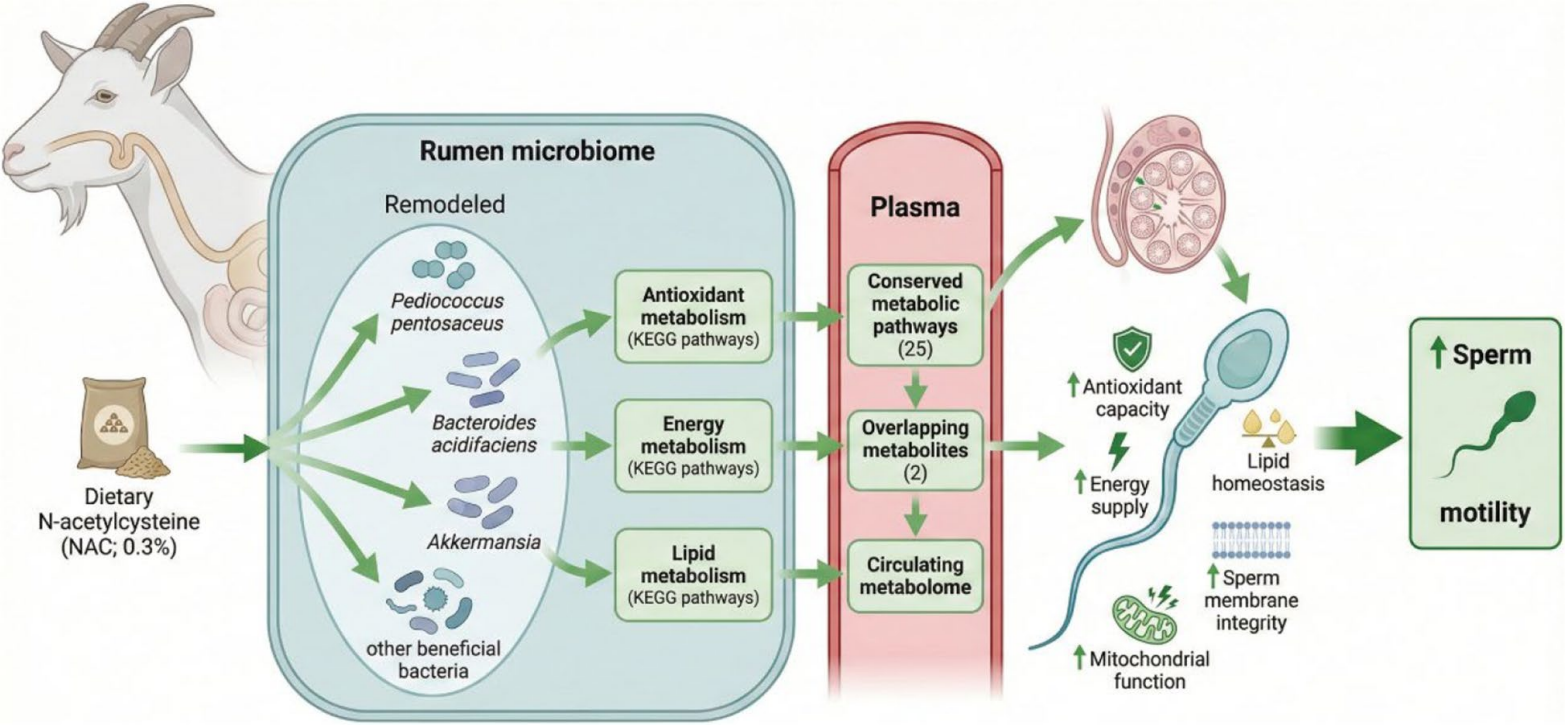

**Supplementary Information:**

The online version contains supplementary material available at 10.1186/s40104-026-01390-2.

## Introduction

Sperm motility is a critical determinant of male fertility [1], directly influencing the success rates of natural conception and assisted reproductive technologies [2, 3]. Notably, a significant proportion of infertility cases in humans and subfertility in animals are attributed to asthenozoospermia [4, 5]. Studies show the clinical significance of progressive motility sperm in promoting cervical mucus penetration [6, 7], in vitro fertilisation [8], and early embryo development [9], thereby improving pregnancy [10] and live birth rates [11]. Consequently, strategies to enhance sperm motility are of paramount importance in reproductive biology [12]. Dietary antioxidants, such as L-arginine [13], vitamin E [14], and flaxseed oil [15], have demonstrated efficacy in improving semen quality, particularly sperm motility. Among these antioxidants, N-acetylcysteine (NAC, C_5_H_9_NO_3_S, MW 163.2), a potent thiol antioxidant and a glutathione precursor [16], has been shown to improve sperm parameters in patients with infertility [17, 18]. However, these benefits have been attributed mainly to its direct antioxidant effects on spermatozoa or the reproductive tract.

A paradigm shift is emerging with the recognition of the microbiota–gonad axis, where intestinal microbiota and their metabolites systemically regulate testicular function and semen quality [19, 20]. In ruminants, the rumen microbiome acts as a primary nutrient metabolism hub, with a fermentation capacity 7.14 times that of the hindgut [21], fundamentally influencing host physiology [22, 23]. We hypothesise that the reproductive benefits of dietary compounds might be significantly mediated through this microbial interface. While NAC's antioxidant properties are well-documented, its impact on the rumen microbiome and whether this serves as a conduit for its beneficial effects on sperm motility under normal physiological conditions remain unexplored. This gap is critical, as it overlooks the potential for NAC to act as a microbe-targeted supplement rather than a direct therapeutic agent.

Recent studies increasingly indicate that the rumen microbiota is closely associated with reproductive performance in ruminants. It has been reported that supplementation with *Moringa oleifera* leaf extract can increase the abundance of fiber-degrading bacteria (e.g., members of the genus *Treponema* and *Fibrobacter*) while decreasing the relative abundance of *Prevotella*, thereby improving sperm concentration, motility, and viability in goats [24]. In sheep showing marked inter-individual variation in sperm motility, the rumen microbial composition also differs accordingly; for example, the abundances of *Ruminococcus*, *Quinella*, and *Lactobacillus* vary in association with motility differences [25]. Mechanistically, perturbations of the rumen microbiota have been suggested to disrupt endocrine homeostasis, including cortisol and testosterone, accompanied by reduced levels of metabolites in rumen fluid and seminal plasma, ultimately leading to impaired sperm motility [26]. Moreover, multi-omics association analyses further demonstrate that *Ruminococcus* and *Quinella* are positively correlated with sperm motility [25], whereas *Prevotella* and *Enterococcus faecalis* are negatively correlated [24, 27]. Collectively, these findings suggest that dietary interventions that favorably reshape the microbial community could be a novel strategy for enhancing fertility [28–30]. NAC, with its reducible thiol group [31], could potentially create a unique ruminal redox environment that selectively enriches for beneficial microbes, initiating a cascade of metabolic events that ultimately support sperm function. Nevertheless, a direct link between NAC, the rumen microbiome, and its metabolic output leading to improved sperm motility has not been established.

Therefore, this study was designed to transcend the conventional view of NAC as a direct antioxidant and to investigate a novel “rumen–plasma–sperm” axis in goats. By employing an integrated multi-omics approach (16S rRNA gene sequencing and metabolomics), we aim to: 1) determine the optimal dietary NAC level for improving sperm motility; 2) assess whether NAC remodels the rumen microbiome and its functional metabolic pathways; and 3) trace the flow of microbial-derived metabolites from the rumen to the systemic circulation and establish their correlation with, and functional contribution to, enhanced sperm motility. This study therefore aims to investigate the potential of the rumen microbiome as a central therapeutic target for nutritional interventions aimed at improving male fertility in ruminants.

## Materials and methods

### Animal diet and experimental design

#### NAC feeding in goats

All animal (Qianbei Ma goats and mice) experiments were approved by the Ethics Committee of Guizhou University (No. EAE-GZU-2023-E078). Using a single-factor randomised trial design, 36 healthy male Qianbei Ma goats aged 18–20 months were randomly divided into four groups: control, 0.1% NAC, 0.3% NAC, and 0.5% NAC groups (*n* = 9). All goats were individually fed in 36 pens, each measuring 2 m × 2 m. No significant differences in sperm motility (total motility and progressive motility) were observed among the groups (*P* > 0.05; Table S1). NAC (≥ 94%, No: 20230310, Hebei Huayang Biological Technology Co., Ltd., Hebei, China) was supplemented based on dry matter intake, which was calculated as 3% of body weight (Table S2). The concentrate-to-roughage ratio was maintained at 3:7 (w/w). NAC was mixed with concentrate and 1 kg of roughage in each meal before feeding the goats. The remaining roughage was provided after the mixed feed was consumed. Goats were fed twice daily at 09:00 and 17:00, with ad libitum access to water and mineral salt blocks. A 14-day pre-feeding adaptation period preceded semen collection on d 10 and d 12 for quality analysis. The formal feeding period lasted 84 d, with semen collection on d 77 and d 83. Based on semen quality results from the feeding period, rumen fluid and plasma were collected from the control and the best sperm motility groups on d 84. The experimental goats were housed and raised at Fuxing Animal Husbandry Co., Ltd., Xishui County, Guizhou, China. Diets were formulated according to the nutritional requirements for goats (NRC, 2007 [32]; Table S3).

#### Metabolite verification in mice

Sixty-three 8-week-old male SPF grade C57BL/6 J mice were purchased from SPF (Beijing) Biotechnology Co., Ltd. and randomly divided into three groups (*n* = 21). The mice were fed in a controlled environment with a temperature of 22 ± 2 °C, a relative humidity of 40%–60% and a 12-h light/dark cycle. Feed and water were provided ad libitum, and the animals were allowed to acclimate before the start of the gavage experiment. Mice were treated by gavage for 35 d as follows: blank (0), sodium benzoate (400 mg/kg/d), and N-methyl-L-asparagine (300 mg/kg/d) groups. The animals were sacrificed by cervical dislocation, and epididymal tail tissues were collected from each group for sperm separation. During the gavage experiment, some mice died owing to gavage-related stress or injury. Therefore, the final number of animals included in the statistical analyses varied by outcome and is indicated in the corresponding figure legends.

### Sample collection

#### Rumen fluid collection

On the final day of the experiment, rumen fluid samples were collected from the control and 0.3% NAC groups. Before morning feeding, rumen fluid was extracted using a rumen cannula. The first 50 mL was discarded, and the remaining fluid was filtered through four layers of gauze. The filtrate was aliquoted into 15-mL centrifuge tubes, flash-frozen in liquid nitrogen, stored on dry ice, and transferred to a −80 °C freezer.

#### Plasma and serum collection

Blood samples were collected at 08:00 from fasting goats in the control and 0.3% NAC groups (nine goats per group). Venous blood was collected via jugular venipuncture using sodium heparin-coated tubes (for plasma). In parallel, additional blood was collected into serum separation tubes (without anticoagulant) to obtain serum. The samples were centrifuged at 3,000 × *g* for 10 min, after which the supernatant layers were promptly aliquoted into pre-labelled 1.5-mL cryovials. The serum and plasma were flash-frozen in liquid nitrogen, stored on dry ice, and transferred to a −80 °C freezer.

#### Fresh semen collection and sperm separation

Semen samples were aseptically harvested from goats using standardised artificial vagina protocols, diluted 20 times, and stored under the same conditions. Owing to the 350 km distance (approximately 3 h drive) between the testing site and the laboratory, semen samples were transported in a car refrigerator. During transportation, the temperature was maintained at 17 °C for 1 h and 4 °C for 2 h.

Differential centrifugation was employed to separate sperm and seminal plasma. Briefly, semen was gently mixed with preheated phosphate buffer saline (PBS) in a 1:2 (v/v) ratio in a 15-mL centrifuge tube and centrifuged at 800 × *g* for 20 min at room temperature (20–25 °C). The sperm pellet was collected, and live/dead cells were separated. The sperm cells were then washed three times with PBS by centrifugation at 800 × *g* for 10 min at room temperature.

#### Mouse epididymal tail sperm separation

The mice were sacrificed by cervical dislocation, and the epididymal tail tissue was removed and put into 400 µL semen diluent. The tissue was finely minced and incubated at 37 °C for 15 min. After incubation, the mixture was filtered through a 70-µm sieve to obtain the sperm-containing diluent. The diluent was centrifuged at 800 × *g* for 10 min at room temperature (20–25 °C) to separate epididymal sperm from epididymal tissue fluid. The separated sperm were resuspended in a fresh semen diluent.

### Semen quality test and analysis

#### Sperm motility and density test

Sperm density and motility parameters—including total motility, progressive motility, circular motility, fast motility, slow motility, non-progressive motility, and immotility—were assessed using computer-assisted sperm analysis (AndroVision^®^, 12500/0000).

#### Assessment of sperm deformity rate

Sperm count and total sperm number, including defects in the caput, corpus, and cauda, were observed under a microscope after staining with Quick Sperm Stain (D029-1; Nanjing Jiancheng Bioengineering Institute, Nanjing, China). The sperm deformity index (SDI) was determined by dividing the total number of morphological abnormalities by the total sperm count according to standard protocols from instruction manual.

#### Assessment of DNA damage in spermatozoa

DNA damage in spermatozoa was assessed using γ-H2AX immunofluorescence (Beyotime Biotechnology, C2035S) with a DNA damage assay kit. The procedure was as follows: semen aliquots (4 μL) were diluted in PBS at a ratio of 1:50, centrifuged at 1,000 × *g* for 10 min, and fixed with 4% paraformaldehyde for 10 min. After washing with PBS, the pellets were resuspended in 100 μL permeabilisation buffer. Smears were air-dried, blocked with 5% bovine serum albumin for 20 min, then incubated with anti-γ-H2AX primary antibody (1:200, 4 °C overnight). Following three PBS washes, Alexa Fluor 488-conjugated secondary antibody was added. After washing, the smears were incubated with DAPI nuclear staining solution at 23 °C for 6 min. Subsequently, an appropriate amount of anti-fluorescence quenching sealing solution was added, and coverslips were applied to seal the samples. The cells were observed under a C2 confocal laser microscope (Nikon Precision (Shanghai) Co., Ltd., Shanghai, China ), and green fluorescence intensity was calculated using ImageJ.

#### Sperm mitochondrial membrane potential

The mitochondrial polarization status of spermatozoa was quantitatively analysed using JC-1 fluorochrome-based cytometry (M8650, Beijing Solarbio Science & Technology Co., Ltd., China). Briefly, 30 μL aliquots of semen specimens were homogenised in 500 μL semen extender, followed by the addition of an equivalent volume of JC-1 staining solution. The sperm suspension was subjected to a standardised staining protocol involving 20 min incubation in a preheated water bath maintained at 37 °C. After incubation, phase separation was achieved by centrifugation (600 × *g*, 5 min, 4 °C) to sediment the spermatozoa, followed by supernatant removal. The harvested sperm underwent two successive washes with JC-1 equilibration buffer (1 ×) to eliminate unbound fluorophores, with final reconstitution in 500 μL fresh buffer prior to cytometric analysis. Mitochondrial membrane potential (MMP) was assessed using a flow cytometer (Becton, Dickinson and Company, Canto II plus), and the proportions of high- and low-membrane-potential sperm were analysed using NovoExpress (version 1.2.5).

#### Determination of antioxidant capacity

Antioxidant assays were performed using commercial kits (Shanghai Enzyme-linked Biotechnology Co., Ltd.) with the following specifications: total antioxidant capacity (T-AOC, #ml094999), catalase-CAT (#ml095173), glutathione peroxidase-GSH-Px (#ml025571), superoxide dismutase-SOD (#ml076325), and reagents for assessing Malondialdehyde-MDA (BC0025) were obtained from Beijing Solarbio Science & Technology Co., Ltd. Assays were conducted according to manufacturer protocols.

#### Evaluation of sperm plasma membrane integrity

The structural integrity of the sperm plasma membrane was evaluated using a hypo-osmotic swelling (HOS) assay kit (G2580, Beijing Solarbio Science & Technology Co., Ltd.). Briefly, 1 mL of hypo-osmotic solution was equilibrated to 37 °C in a sterile tube for 5 min. Then, 0.1 mL of semen was introduced into the solution, mixed gently, and incubated at 37 °C for 60 min. Following incubation, a light microscope was employed to assess sperm morphology. Cells with coiled tails were considered to have intact membranes and were therefore counted as viable.

### Detection of rumen fluid microbial flora

Microbiome analysis involved CTAB-phenol–chloroform DNA extraction followed by electrophoretic integrity checks and NanoDrop quantification. Target-specific primers (Table S4) were amplified, electrophoretically validated (2% agarose), and processed into Illumina-compatible libraries. Denatured with NaOH for single-strand preparation, libraries underwent paired-end sequencing (2 × 250 bp) on NovaSeq 6000 (SP500 kit). Taxonomic assignment via SILVA v138/NT-16S (confidence ≥ 0.7) preceded α-diversity comparisons using Kruskal–Wallis tests (*P* < 0.05), with ASV abundance matrices guiding community structure analysis.

### Metabolome sequencing

Metabolomic profiling was conducted using deuterated internal standard-enhanced LC–MS/MS. Ruminal fluid and plasma aliquots (100 μL) were subjected to protein precipitation via homogenization with 400 μL ice-cold methanol:acetonitrile (1:1) containing isotopic controls. Following centrifugation (13,800 × *g*, 15 min, 4 °C), supernatants were collected and pooled for QC preparation. Chromatographic separation employed a Waters BEH Amide column (2.1 mm × 50 mm, 1.7 μm) on a Vanquish UHPLC-Orbitrap Exploris 120 system with binary mobile phases: (A) 25 mmol/L ammonium acetate/ammonium hydroxide (pH 9.75) and (B) acetonitrile. MS parameters included dual-polarity ESI (+3.8/−3.4 kV), 60 k/15 k resolution (MS1/MS2), and stepped collision energy of 20/30/40 eV. Samples were acquired with a 2-μL injection on a 4 °C autosampler. Metabolite significance thresholds were VIP > 1 and *P* < 0.05 via multivariate analysis.

### Targeted validation of differentially abundant metabolites

Plasma aliquots were homogenised by ice-thawing and vortex-mixing for 30 s, followed by dilution in 250 μL H_2_O. Protein precipitation was performed by adding 1,200 μL prechilled (−40 °C) methanol:acetonitrile (1:1) extraction solvent containing internal standards. The homogenate was subsequently vortex-mixed for 30 s, ultrasonicated in an ice-water bath for 15 min, incubated at −40 °C for 2 h, and centrifuged at 13,800 × *g* for 15 min at 4 °C. The supernatant (1,200 μL) was concentrated via centrifugation to dryness (8 h) and reconstituted in 120 μL acetonitrile:water (6:4) using vortex-ultrasonic dissolution in an ice bath for 30 s. The solution was then centrifuged at 13,800 × *g* for 15 min at 4 °C, and 70 μL of the resulting supernatant was transferred to an LC injection vial. Targeted quantitative detection of L-tyrosine, benzoic acid, and L-tryptophan in plasma was performed using ultrahigh-performance liquid chromatography (Agilent 1290).

### Statistical analysis

Statistical analysis was performed using SPSS (v.20.0, IBM, Chicago, IL, USA). One-way analysis of variance (ANOVA) followed by Tukey's multiple-comparison test was used for multiple-group comparisons. For two-group comparisons, an independent-samples two-tailed Student's *t*-test was applied when equal variances were assumed; otherwise, Welch's t-test was used. Spearman correlation analysis was performed using the Lianchuan Biological Online Cloud Platform (https://www.omicstudio.cn/tool). Graphs were drawn using GraphPad Prism 8 (GraphPad, La Jolla, CA, USA) and Cytoscape 3.10.3 (Cytoscape Consortium, https://cytoscape.org). Data are expressed as mean ± standard error of the mean (SEM). Statistical significance was defined as ^*^P < 0.05, ^**^P < 0.01, and ^***^P < 0.001.

## Results

### Effects of NAC feeding on semen quality

**Fig. 1:**
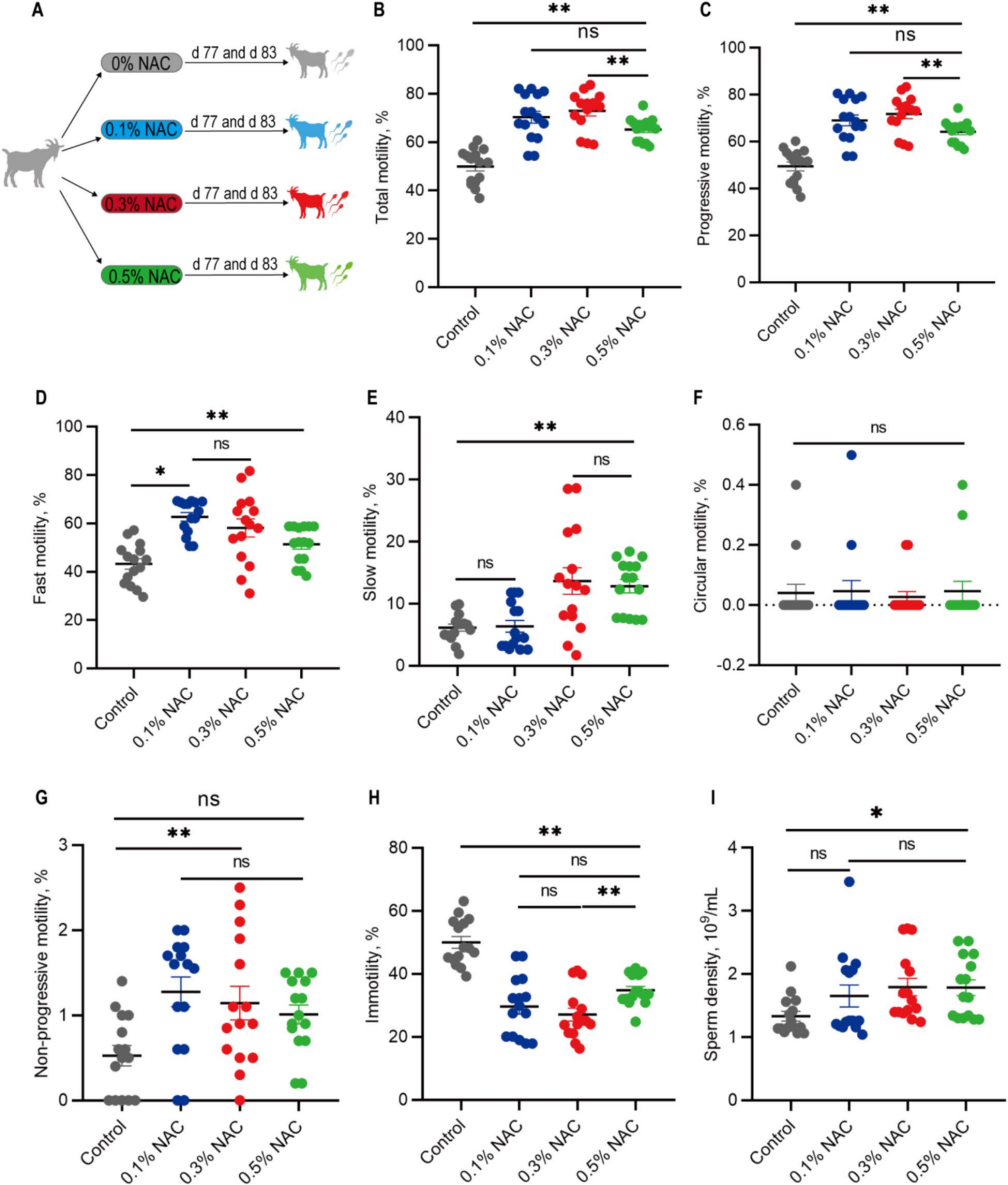
Effects of NAC on the sperm motility and density of the Qianbei Ma goats. **A** Schematic diagram of the experiment on the effects of NAC on sperm motility and density. **B**‒**I** Effects of different concentrations of NAC supplemented in the diet on total motility (**B**), progressive motility (**C**), fast motility (**D**), slow motility (**E**), circular motility (**F**), non-progressive motility (**G**), immotility (**H**) and sperm density (**I**). *n* = 15 (7 from d 77, 8 from d 83). ^*^P < 0.05, ^**^P < 0.01, “ns” indicates no significant difference

The effects of NAC supplementation on semen quality were evaluated by analysing sperm motility and density at varying NAC concentrations (Fig. 1A). Among the sperm motility indicators, total and progressive motility were significantly higher in the 0.1%, 0.3%, and 0.5% NAC groups than in the control group (*P* < 0.01). The 0.3% NAC group showed a trend toward higher motility than the 0.1% NAC group (0.05 < *P* < 0.10) and significantly exceeded the 0.5% NAC group (*P* < 0.01; Fig. 1B and C). Fast motility analysis revealed that the 0.3% and 0.5% NAC groups exhibited significantly higher motility than those in the control group (*P* < 0.01). The 0.1% NAC group also had significantly higher levels than the control group (*P* < 0.05); however, no significant difference was observed between the 0.1% and 0.3% NAC groups (*P* > 0.05). Notably, the 0.1% and 0.3% NAC groups had significantly higher levels than the 0.5% NAC group (*P* < 0.01; Fig. 1D). Slow motility analysis revealed that the 0.3% and 0.5% NAC groups showed significantly greater effects than those in the control and 0.1% NAC groups (*P* < 0.01; Fig. 1E), with no significant difference between the 0.3% and 0.5% NAC groups (*P* > 0.05). The circular motility test result showed no significant differences among all the groups (*P* > 0.05; Fig. 1F). Non-progressive motility results showed that the 0.1% and 0.3% NAC groups had significantly higher levels than those in the control group (*P* < 0.01; Fig. 1G). Immotility results showed that the control group had significantly higher immotile sperm activity than that in the 0.1%, 0.3%, and 0.5% NAC groups (*P* < 0.01). Additionally, the activity in the 0.5% NAC group was significantly higher than that in the 0.3% NAC group (*P* < 0.01; Fig. 1H). Sperm density in the 0.3% and 0.5% NAC groups was significantly higher than that in the control group (*P* < 0.05; Fig. 1I). Overall, NAC supplementation enhanced sperm motility and density, with the 0.3% NAC group showing the greatest effect. Therefore, the 0.3% NAC group was selected for further experiments.

### Effects of NAC on sperm functional parameters

**Fig. 2:**
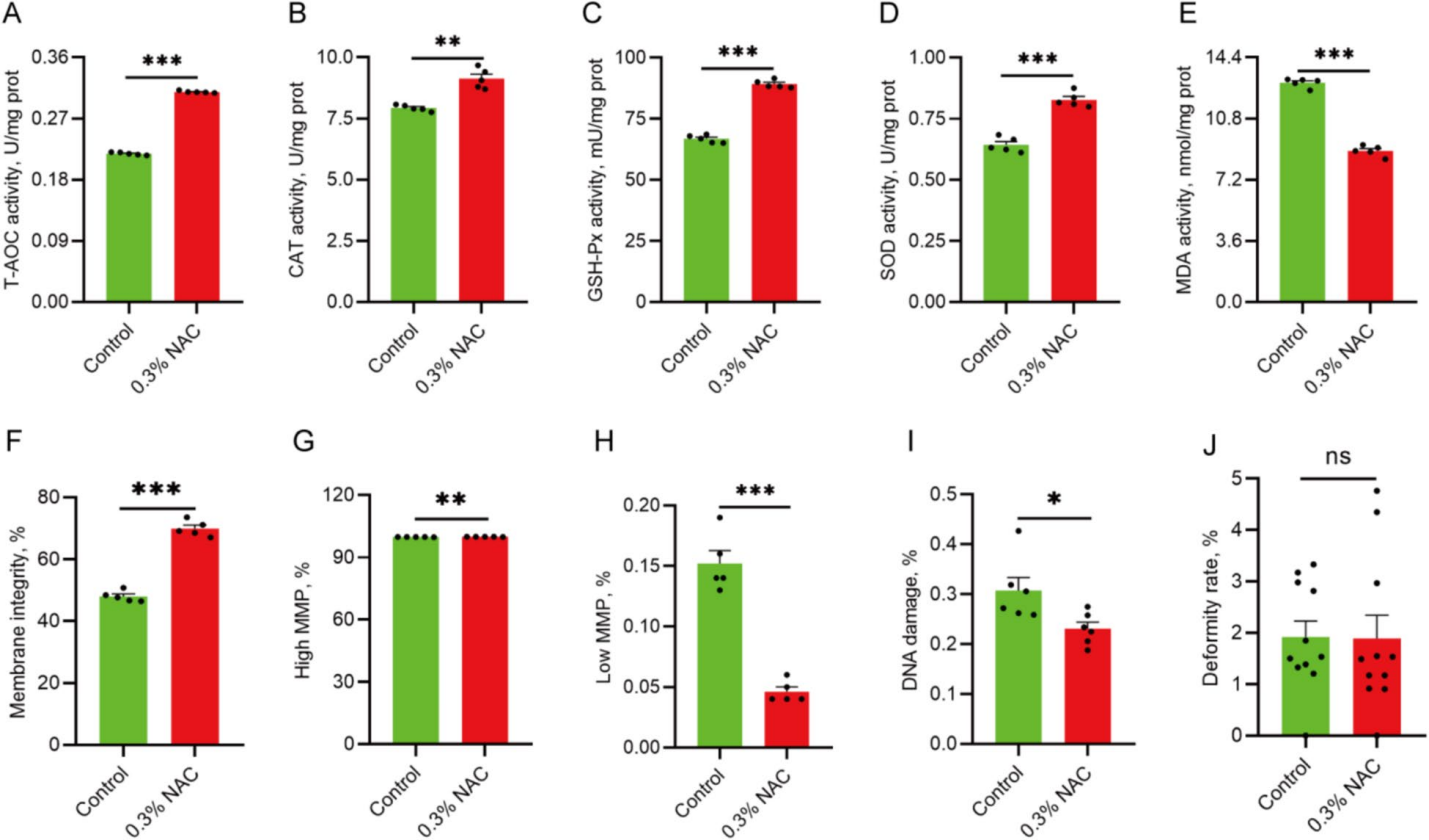
Effect of dietary NAC supplementation on the sperm functional parameters of the Qianbei Ma goats. **A**–**E** Effects of dietary NAC supplementation on the sperm T-AOC (**A**), CAT (**B**), GSH-Px (**C**), SOD (**D**), MDA (**E**) (*n* = 5). **F** Membrane integrity of sperm (*n* = 5). **G **and** H** Proportion of high (**G**) and low (**H**) membrane potential sperm (*n* = 5). **I** Proportion of DNA damage (*n* = 6). **J** Sperm deformity rate (*n* = 5). ^*^P < 0.05 indicates significant differences, ^**^P < 0.01 indicates extremely significant differences, ^***^P < 0.001 indicates highly significant differences, “ns” indicates no significant difference

To further explore the mechanism by which NAC enhances sperm motility, sperm antioxidant indicators, plasma membrane integrity, mitochondrial membrane potential, DNA damage, and deformity rate were evaluated after supplementing with NAC. The results showed that compared with the control group, supplementing 0.3% NAC in the diet significantly increased the levels of T-AOC, CAT, GSH-Px, SOD in sperm(*P* < 0.01; Fig. 2A–D), and decreased the level of MDA (*P* < 0.001; Fig. 2E). Supplementation with NAC enhanced sperm plasma membrane integrity (*P* < 0.001; Fig. 2F), increased the proportion of sperm with high membrane potential (*P* < 0.01; Fig. 2G, Fig. S1A), and decreased the proportion of low membrane potential sperm (*P* < 0.001; Fig. 2H, Fig. S1B), DNA damage (*P* < 0.05; Fig. 2I, Fig. S1C), and sperm deformity rate (*P* > 0.05; Fig. 2J). Overall, NAC supplementation positively influenced functional parameters that support sperm motility.

### Effects of NAC feeding on the rumen microflora and functional profiles

#### Overview of rumen microbial composition

**Fig. 3:**
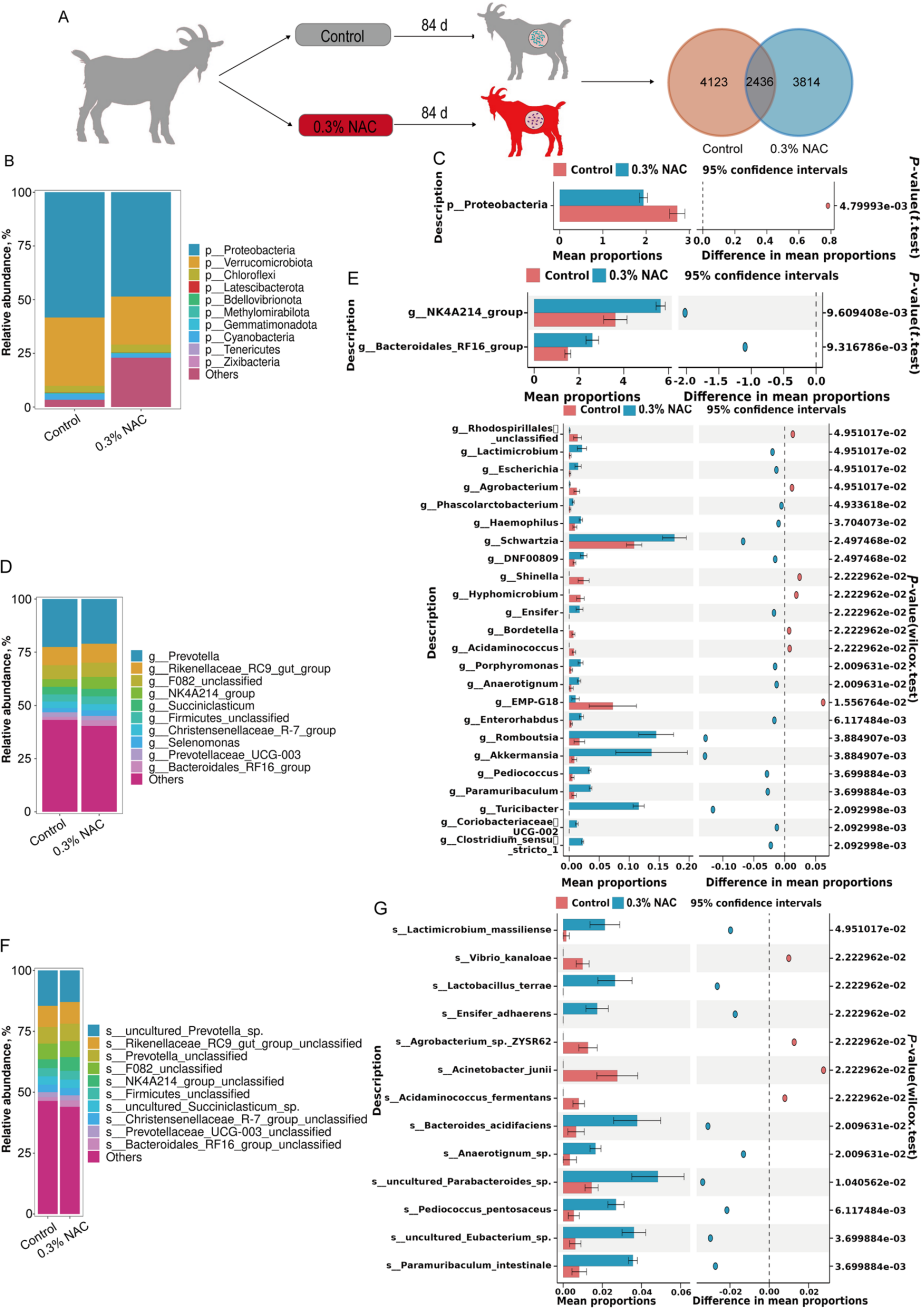
Effects of dietary NAC supplementation on the rumen microbiota of Qianbei Ma goats. **A** Schematic diagram of the effect of NAC on the rumen microbiota (Venn diagram: ASV distribution). **B** Relative abundance of the top 10 bacterial phyla. **C** Rumen bacterial phylum with significant differences. **D** Relative abundance of the top 10 bacterial genera. **E** Rumen bacterial genus with significant differences. **F** Relative abundance of the top 10 bacterial species. **G** Rumen bacterial species with significant differences

To analyse the effects of NAC supplementation on rumen microflora, 16S rRNA gene sequencing was performed on rumen fluid samples from the control and 0.3% NAC groups. The results showed that the total number of amplicon sequence variants (ASV) in the control and 0.3% NAC groups was 10,373, with 4,123 being unique to the control group, 3,814 to the 0.3% NAC group, and 2,436 shared between both groups (Fig. 3A). Alpha diversity analysis revealed no significant differences in the Shannon, Simpson, Chao1, Good’s coverage, or Pielou_e indices between the control and 0.3% NAC groups (*P* > 0.05; Fig. S2A). However, these indices exhibited varying degrees of increase or decrease (Fig. S2A). The principal component analysis showed partial overlap and distinct separation between the two groups (Fig. S1B). At the phylum level, the top 10 phyla with relative abundance are displayed in Fig. 3B. Compared with that in the control group, the relative abundance of Proteobacteria in the 0.3% NAC group decreased significantly (*P* < 0.05; Fig. 3C), while no significant differences were observed in other phyla (*P* > 0.05). At the genus level, the top 10 bacterial genera with relative abundance are displayed in Fig. 3D. Statistical analysis of metagenomic profiles (STAMP) revealed 19 bacterial genera with significantly increased relative abundance, including *Turicibacter, Clostridium_sensu_stricto_1*, and *Akkermansia* (*P* < 0.05; Fig. 3E)*.* Conversely, the relative abundances of seven bacterial genera, including *EMP-G18*, *Shinella*, and *Hyphomicrobium*, significantly decreased (*P* < 0.05; Fig. 3E). At the species level, the top 10 species with relative abundance are displayed (Fig. 3F). Following NAC supplementation STAMP showed a significant increase in the abundance of nine bacterial species, including *Paramuribaculum intestinale*, *uncultured Eubacterium* sp., and *Pediococcus pentosaceus* (*P* < 0.05; Fig. 3G). However, the abundance of four bacterial species, including *Acinetobacter junii*,* Agrobacterium* sp., *ZYSR62*, and *Vibrio kanaloae*, significantly decreased (*P* < 0.05; Fig. 3G). The LEfSe analysis showed that seven different classification levels of bacteria were found between the control and 0.3% NAC groups, with LDA values in both groups higher than 3 (Fig. S2C and D). These results showed that NAC supplementation changed the rumen microenvironment of Qianbei Ma goat.

#### Functional profiles of rumen microbiome

**Fig. 4:**
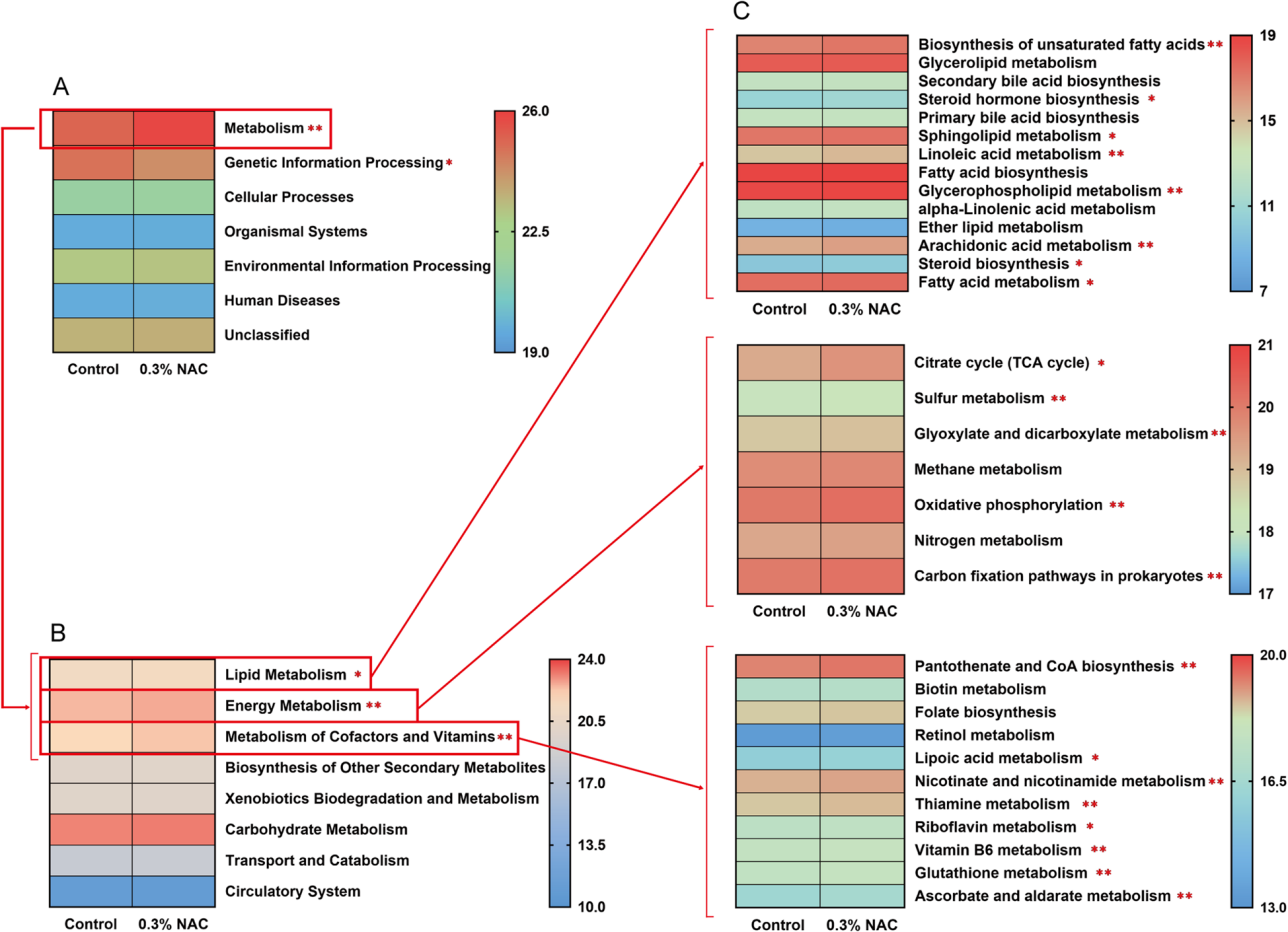
Difference in functional parameters of the rumen microbiome. **A**–**C** Relative abundance of function KEGG pathway, level 1 (**A**), level 2 (top 8 of metabolism) (**B**), level 3 (top 14 of “Lipid Metabolism”, top 7 of “Energy Metabolism” and top 11 of “Metabolism of Cofactors and Vitamins”) (**C**). KEGG pathways were compared using linear discriminant analysis effect size (LEfSe), with LDA score > 2 and *P* < 0.05 being considered as significantly different. ^*^LDA score > 2 and *P* < 0.05, ^**^LDA score > 2 and *P* < 0.01

For bacterial genera or strains that differed significantly, functional analysis was conducted using the Kyoto Encyclopedia of Genes and Genomes (KEGG) atlas, and the results showed significant separation between the control and 0.3% NAC groups (Fig. 4). Specifically, the analysis of the KEGG level 1 pathway identified the main categories of differences in rumen microbiota between the control and the 0.3% NAC groups: Metabolism (25.53%), Genetic Information Processing (24.31%), Cellular Processes (21.54%), Organismal Systems (19.63%), Environmental Information Processing (23.13%), Human Diseases (19.65%), Unclassified (23.56%). Notably, “metabolism” exhibited more enrichment in the rumen microbiome of the 0.3% NAC group (25.62%) than that in the control group (25.45%). Similarly, “Genetic Information Processing” showed more enrichment in the rumen microbiome of the 0.3% NAC group (24.35%) than that in the control group (24.28%) (LDA > 2, *P* < 0.05; Fig. 4A, Table S5). At the KEGG level 2, within metabolic pathways beneficial for sperm motility regulation, the rumen microbiota in the 0.3% NAC group demonstrated significant enrichment in the subcategories of “Lipid Metabolism”, “Energy Metabolism”, and “Cofactor and Vitamin Metabolism” (LDA > 2, *P* < 0.05; Fig. 4B, Table S5). At KEGG level-3, enrichment was observed in the 0.3% NAC group within the “Lipid Metabolism”, “Energy Metabolism”, and “Metabolism of Cofactors and Vitamins” pathways. Specifically, eight “Lipid Metabolism” pathways (biosynthesis of unsaturated fatty acids, steroid hormone biosynthesis, sphingolipid metabolism, linoleic acid metabolism, glycerophospholipid metabolism, arachidonic acid metabolism, steroid biosynthesis, and fatty acid metabolism), five “Energy Metabolism” pathways (citrate cycle [TCA cycle], sulfur metabolism, glyoxylate and dicarboxylate metabolism, oxidative phosphorylation, and carbon fixation pathways in prokaryotes) and eight “Metabolism of Cofactors and Vitamins” pathways (pantothenate and CoA biosynthesis, lipoic acid metabolism, nicotinate and nicotinamide metabolism, thiamine metabolism, riboflavin metabolism, vitamin B_6_ metabolism, glutathione metabolism, ascorbate, and aldarate metabolism) were all significantly enriched (LDA > 2, *P* < 0.05; Fig. 4C, Table S5).

#### Relationship among phenotype, rumen microbiome, and microbial function

**Fig. 5:**
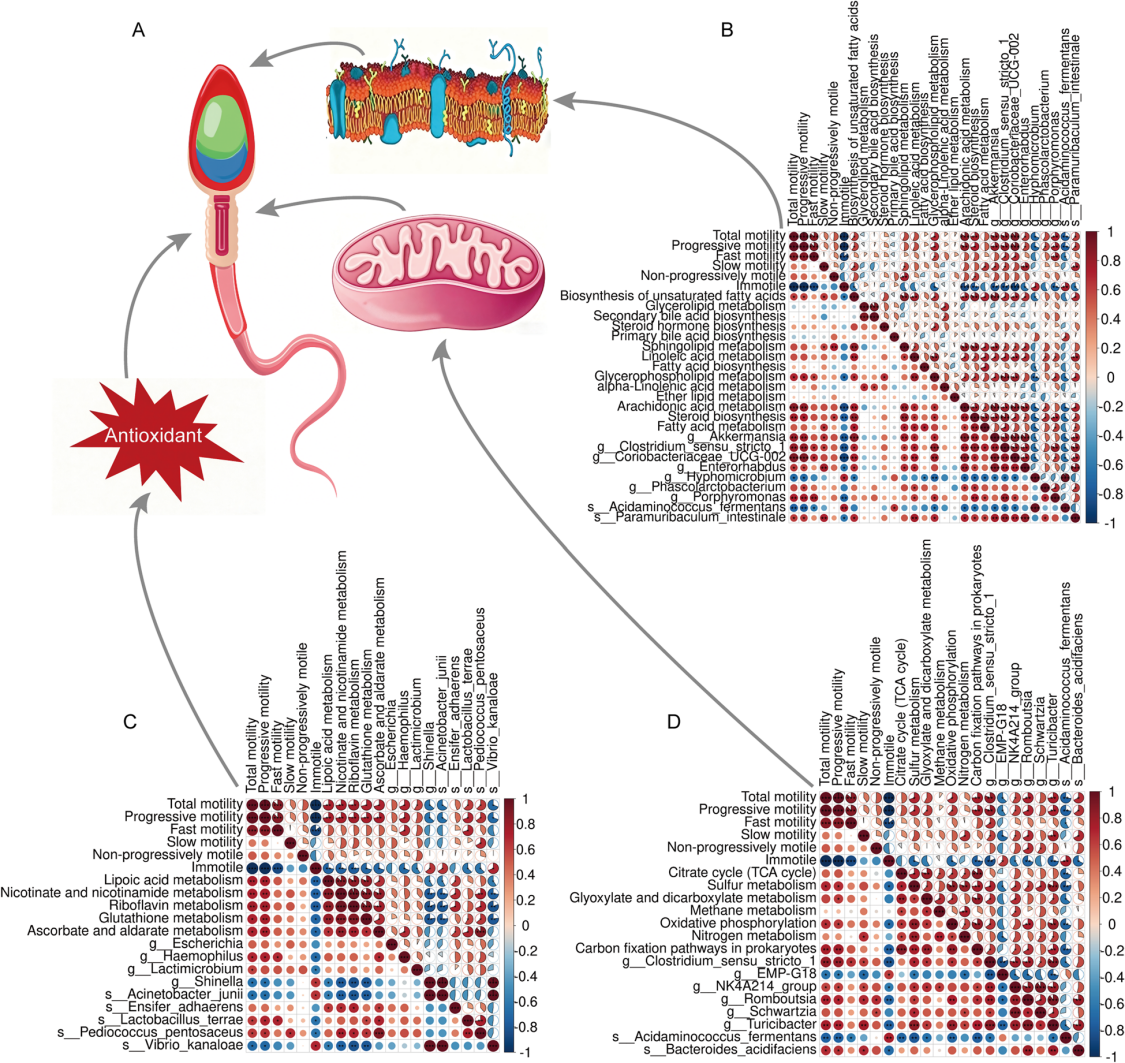
Relationship among phenotype, rumen microbiome and microbial function. **A** “Lipid Metabolism”, “Energy Metabolism” and “Metabolism of Cofactors and Vitamins (antioxidant)” mechanism diagram of action to sperm. **B** Relationship among sperm motility, rumen microbiome and microbial function in “lipid metabolism” pathway. **C** Relationship among sperm motility, rumen microbiome and microbial function in “Metabolism of Cofactors and Vitamins (antioxidant)” pathway. **D** Relationship among sperm motility, rumen microbiome and microbial function in “Energy Metabolism” pathway. Red indicates a positive correlation, blue indicates a negative correlation, and darker colors indicate stronger correlations. ^*^P < 0.05 indicates significant differences, ^**^P < 0.01 indicates extremely significant differences, ^***^P < 0.001 indicates highly significant differences

Correlations were established among the level 3 pathways of “Lipid Metabolism”, “Energy Metabolism”, and “Metabolism of Cofactors and Vitamins”. The genera (species) in the pathways and the host phenotype (sperm motility) were identified. The results showed that in the level 3 pathways of “Lipid Metabolism”, *Akkermansia*, *Clostridium *sensu stricto* 1*, *Coriobacteriaceae* UCG-002, *Enterorhabdus*, *Hyphomicrobium, Phascolarctobacterium*, *Porphyromonas*, *Acidaminococcus fermentans,* and *Paramuribaculum intestinale* were significantly positively correlated (seven bacteria) or negatively correlated (two bacteria) with total motility and progressive motility (*P* < 0.05; Fig. 5A and B). In the level 3 pathways of “Metabolism of Cofactors and Vitamins”, *Shinella*, *Haemophilus*, *Acinetobacter junii*, *Lactobacillus terrae*, *Pediococcus pentosaceus*, and *Vibrio kanaloae* were significantly positively correlated (three bacteria) or negatively correlated (three bacteria) with total motility and progressive motility (*P* < 0.05; Fig. 5A and C). Notably, rumen bacteria, particularly *Pediococcus pentosaceus* and *Lactobacillus terrae* in the “Metabolism of Cofactors and Vitamins” pathway, have a specific role in antioxidant performance. This suggests that microbial regulation of cofactors and vitamin metabolism contributes to antioxidant activity and thus supports sperm motility. In the level 3 pathway of “Energy Metabolism”, *Clostridium *sensu stricto* 1*, EMP-G18, NK4A214 group, *Romboutsia*, *Turicibacter*, *Acidaminococcus fermentans*, and *Bacteroides acidifaciens* were significantly positively correlated (five bacteria) or negatively correlated (two bacteria) with total motility and progressive motility (*P* < 0.05; Fig. 5A and D). Collectively, our results identified the rumen microbiota associated with sperm motility and demonstrated that NAC-induced changes in this microbiota are linked to its regulation, pointing to a novel connection between rumen microbiota and reproductive performance.

### NAC affects metabolite changes in rumen fluid

**Fig. 6:**
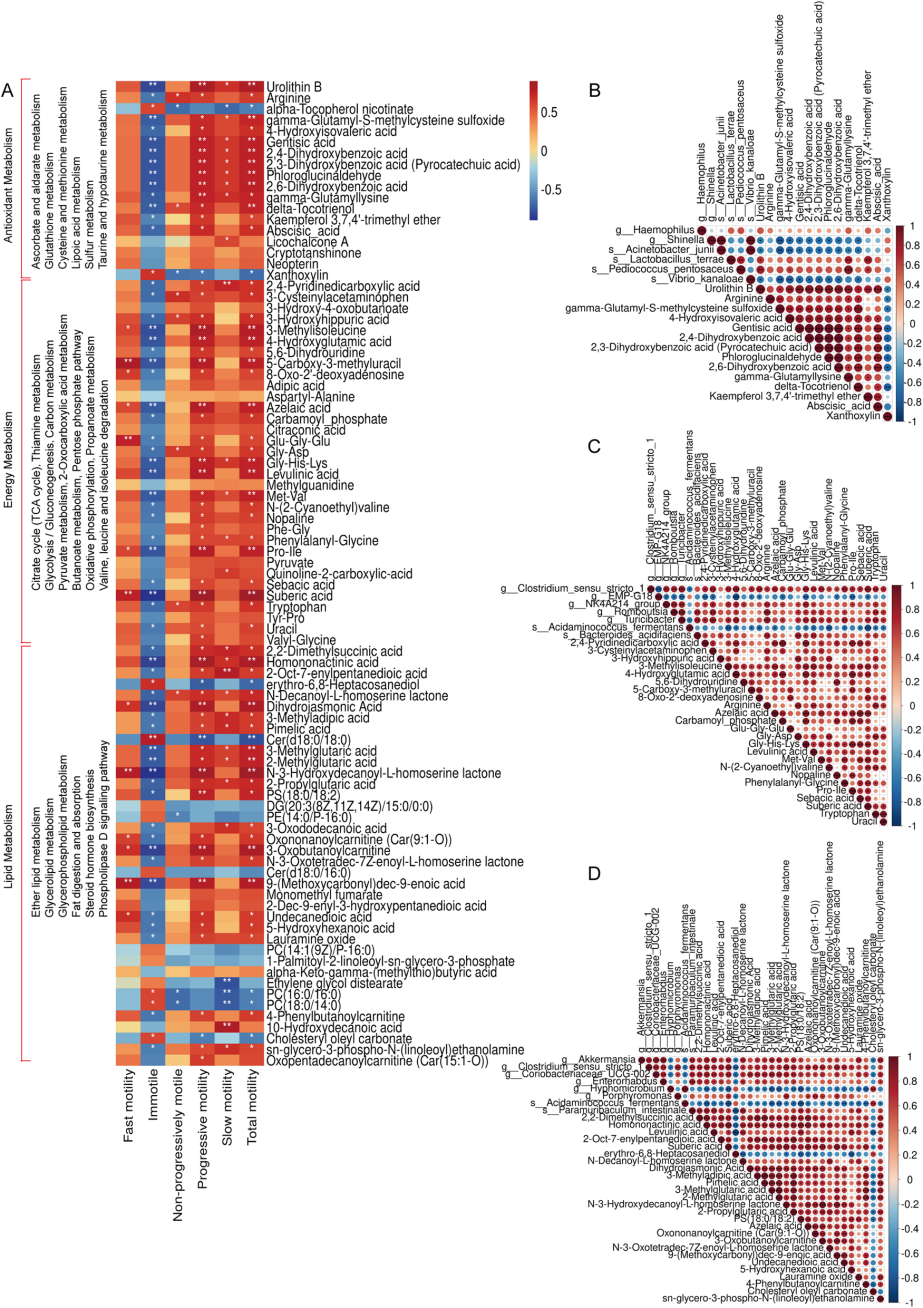
Effects of dietary NAC supplementation on metabolic profiles in rumen fluid. **A** Correlation between different metabolites in the functional categories of antioxidant, energy, lipid metabolism of rumen fluid and sperm motility. **B**–**D** Correlations between rumen microbiota, metabolites and pathway associated with sperm motility in the antioxidant (**B**), energy (**C**), and lipid (**D**) metabolism pathways. ^*^P < 0.05 indicates significant differences, ^**^P < 0.01 indicates extremely significant differences, and ^***^P < 0.001 indicates highly significant differences

Rumen metabolome sequencing revealed a clear difference between the two groups (Fig. S3A), and the model was stable (Fig. S3B). A comparative metabolite analysis revealed 244 upregulated and 39 downregulated metabolites (Fig. S3C). Corresponding to the classification of rumen microbiota metabolic functions, we divided rumen fluid metabolic pathways into three major categories: “Antioxidant”, “Energy Metabolism”, and “Lipid Metabolism”. Twenty-three KEGG pathways were classified, including 6 “Antioxidant”, 11 “Energy Metabolism”, and 6 “Lipid Metabolism” (*P* < 0.01; Fig. 6A). Correlation analysis with sperm motility revealed significant associations for metabolites across three metabolic categories. In the antioxidant category, 14 metabolites were significantly correlated with both total and progressive motility, among which 13 were positively correlated and 1 was negatively correlated (*P* < 0.05; Fig. 6A). Within “Energy Metabolism” pathway, 23 metabolites showed significant positive correlations with total and progressive motility (*P* < 0.05; Fig. 6A). Additionally, 24 metabolites in “Lipid Metabolism” were significantly correlated with total and progressive motility, among which 21 were positively correlated and 3 were negatively correlated (*P* < 0.05; Fig. 6A). These results classify them as rumen metabolites related to sperm motility, further demonstrating the mutual evidence of functional and metabolic consistency of microbial communities in rumen fluid.

We performed a joint analysis of sperm motility-related rumen microbiota and metabolites. In “Antioxidant”, it was found that the rumen microbiota *Pediococcus pentosaceus* and *Lactobacillus terrae* enriched in the 0.3% NAC group were positively correlated with the rumen metabolites urolithin B, gamma-glutamyllysine, and kaempferol 3,7,4'-trimethyl ether (*P* < 0.05; Fig. 6B). *Pediococcus pentosaceus* was also positively correlated with 4-hydroxyisovaleric acid (*P* < 0.05; Fig. 6B). In the “Energy Metabolism” pathway, rumen microbiota enriched in the 0.3% NAC group—including *Bacteroides acidifaciens*, *Turicibacter*, *Clostridium *sensu stricto* 1*, NK4A214 group, and *Romboutsia*—were significantly positively correlated with the rumen metabolites arginine, Gly-His-Lys, levulinic acid, and tryptophan (*P* < 0.05; Fig. 6C). Conversely, the rumen microbiota EMP-G18 and *Acidaminococcus_fermentans*, which were enriched in the control group, showed significant negative correlations with the metabolites 4-hydroxyglutamic acid, 5-carboxy-3-methyluracil, 8-oxo-2'-deoxyadenosine, Gly-His-Lys, and suberic acid (*P* < 0.05; Fig. 6C). In the “Lipid metabolism” pathway, rumen microbiota enriched in the 0.3% NAC group—including *Akkermansia*, *Clostridium *sensu stricto* 1*, *Coriobacteriaceae* UCG-002, *Enterorhabdus*, *Porphyromonas*, *Paramuribaculum_intestinale*—showed significant positive correlations with multiple rumen metabolites, including 2,2-Dimethylsuccinic acid, Homononactinic acid, Levulinic acid, 2-Oct-7-enylpentanedioic acid, Suberic acid, N-Decanoyl-L-homoserine lactone, 3-Methyladipic acid, Pimelic acid, 2-Propylglutaric acid, and sn-glycero-3-phospho-N-(linoleoyl)ethanolamine (*P* < 0.05; Fig. 6D). In contrast, the rumen microbiota *Hyomicrobium* and *Acidaminococcus fermentans*, which were enriched in the control group, exhibited highly significant negative correlations with the metabolites suberic acid, 3-Methylglutaric acid, 2-Methylglutaric acid, PS(18:0/18:2) and 4-Phenylbutanoylcarnitine (*P* < 0.01; Fig. 6D).

### Effects of NAC on the plasma metabolome

**Fig. 7:**
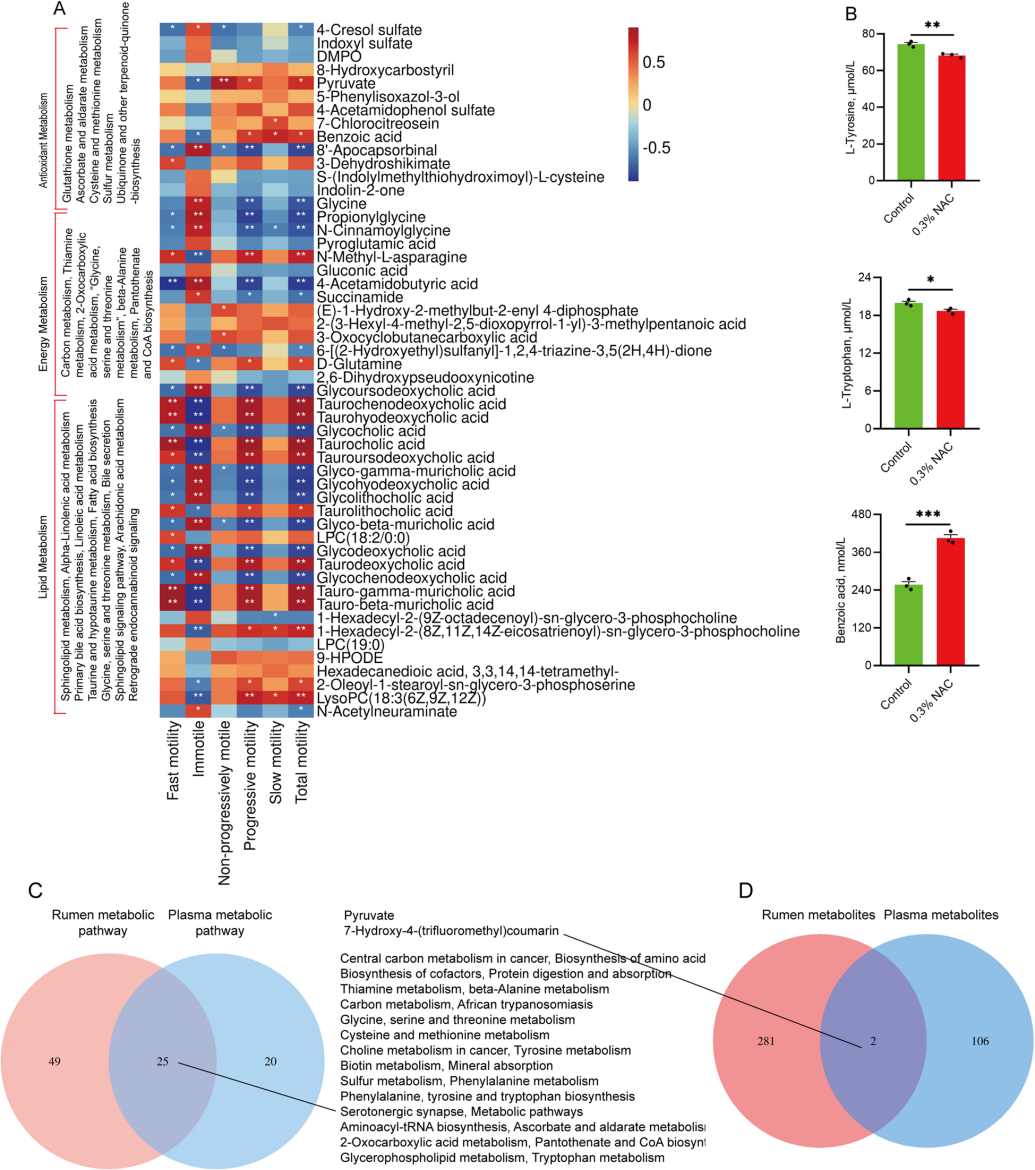
Effects of dietary NAC supplementation on metabolic profiles in plasma. **A** Correlation between different metabolites in the functional categories of antioxidant, energy, lipid metabolism of plasma and sperm motility. **B** Quantitative verification of L-tyrosine, and L-tryptophan, benzoic acid (*n* = 3). **C** Common metabolic pathways in rumen fluid and plasma. **D** Common metabolites in rumen fluid and plasma. ^*^P < 0.05 indicates significant differences, ^**^P < 0.01 indicates extremely significant differences, and ^***^P < 0.001 indicates highly significant differences

Plasma metabolome sequencing revealed a clear difference between the two groups (Fig. S4A), and the model was stable (Fig. S3B). A comparative metabolite analysis revealed 488 upregulated and 317 downregulated metabolites (Fig. S4C). Corresponding to the classification of rumen metabolites functions, we divided plasma metabolic pathways into three major categories: “Antioxidant”, “Energy Metabolism” and “Lipid Metabolism”. Twenty KEGG pathways were classified, including five “Antioxidant”, six “Energy Metabolism”, and nine “Lipid Metabolism” (*P* < 0.01; Fig. 7A). The correlation analysis between enriched differential metabolites in pathways and sperm motility showed that metabolites in all three metabolic pathways were significantly associated with sperm motility. In the antioxidant category, 4 metabolites were significantly correlated with both total and progressive motility, among which pyruvate and benzoic acid were positively correlated and 8'-apocapsorbinal, and glycine were negatively correlated (*P* < 0.01; Fig. 7A). Within “Energy Metabolism”, seven metabolites showed significant positive correlations with both motility parameters, among which N-methyl-L-asparagine and D-glutamine were positively correlated and propionylglycine, N-cinnamoylglycine, 4-acetamidobutyric acid, succinamide, and glycoursodeoxycholic acid were negatively correlated (*P* < 0.01; Fig. 7A). Additionally, 18 metabolites in Lipid Metabolism were significantly correlated with total and progressive motility, among which 11 were positively correlated and 7 were negatively correlated (*P* < 0.01; Fig. 7A). Notably, eight metabolites of the taurine binding bile acid family were among the 11 metabolites significantly positively correlated (upregulated) with sperm motility. Among the seven metabolites significantly negatively correlated (downregulated) with sperm motility. These seven are glycine-binding bile acid family metabolites. Furthermore, we compared the shared metabolic pathways and metabolites between rumen fluid and plasma, and found 25 shared metabolic pathways and 2 shared metabolites (Fig. 7C and D). The shared metabolic pathways of rumen fluid and plasma accounted for 33.78% of all pathways in rumen fluid metabolome sequencing and 55.56% of all pathways in plasma metabolome. To verify the reliability of the sequencing data, L-tyrosine, benzoic acid, and L-tryptophan were randomly selected for quantitative analysis, which confirmed the consistency between quantitative data and sequencing results (*P* < 0.05; Fig. 7B).

### Validation of the effect of plasma metabolites on sperm motility by gavage in mice

**Fig. 8:**
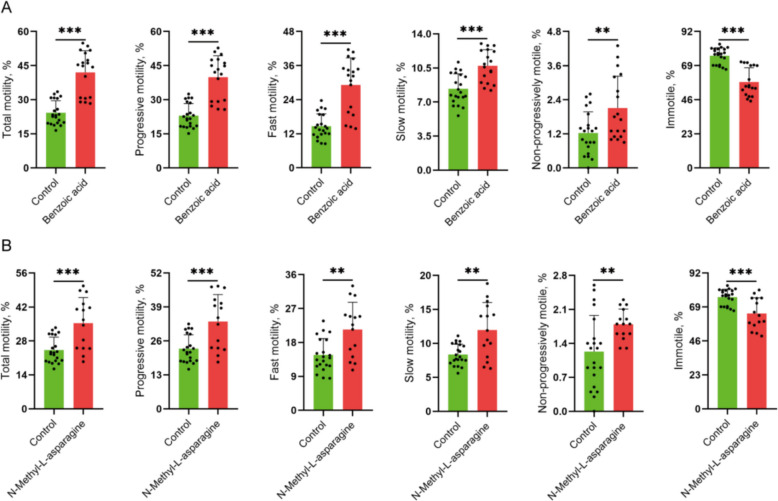
The effects of plasma metabolites benzoic acid and N-methyl-L-asparagine on sperm motility in mice. **A** and **B** Changes in total motility, progressive motility, fast motility, slow motility, non-progressive motility and immotility in the benzoic acid (**A**) and N-methyl-L-asparagine (**B**) gavage groups. *n* = 15, 18 or 21. ^*^P < 0.05 indicates significant differences, ^**^P < 0.01 indicates extremely significant differences, and ^***^P < 0.001 indicates highly significant differences

Results of intragastric administration of the plasma metabolites benzoic acid and N-methyl-L-asparagine in mice demonstrated that both compounds improved sperm motility in the cauda epididymis. Compared with the control group, the benzoic acid group showed increases in total motility (1.73-fold), progressive motility (1.73-fold), fast motility (1.99-fold), slow motility (1.28-fold), and non-progressive motility (1.71-fold), while immotility decreased by 1.31-fold (*P* < 0.01; Fig. 8A). Similarly, the N-methyl-L-asparagine group exhibited increases in total motility (1.46-fold), progressive motility (1.45-fold), fast motility (1.46-fold), slow motility (1.43-fold), and non-progressive motility (1.57-fold), along with a 1.17-fold reduction in immotility (*P* < 0.01; Fig. 8B). These results confirm that metabolites identified in plasma are functionally associated with enhanced sperm motility.

### Identification and variation of core microbiota and metabolites in rumen and plasma transfer chains

**Fig. 9:**
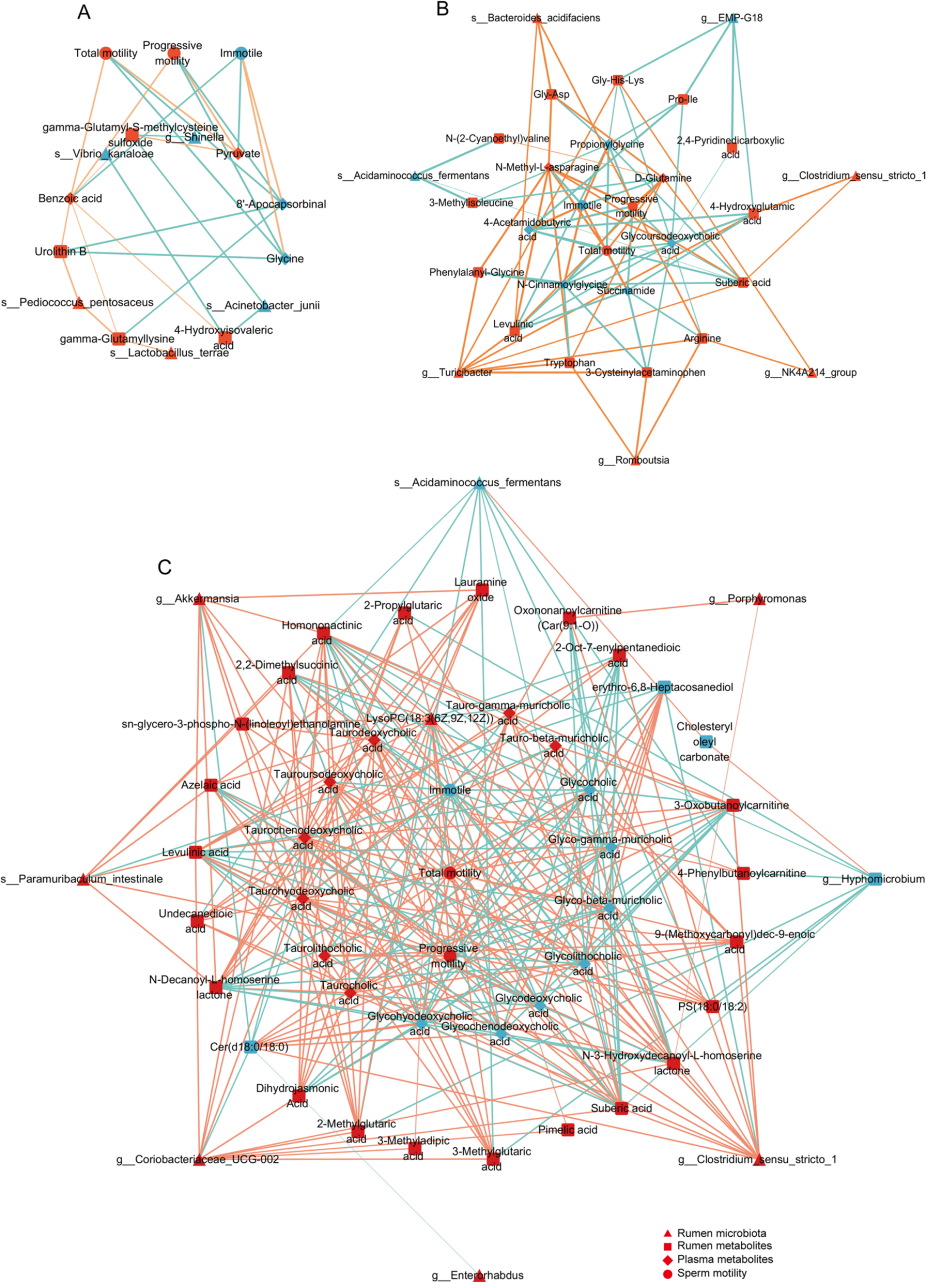
The network relationship between rumen microbiota, rumen metabolites, plasma metabolites, and host phenotype. **A** The network between metabolites, metabolites, and host phenotypes in the pathway related to antioxidant metabolism. **B** The network between metabolites, metabolites, and host phenotypes in the pathway related to energy metabolism. **C** The network between microorganisms, metabolites, and host phenotypes in the pathway related to lipid metabolism. The red nodes represent upward adjustments, while the blue nodes represent downward adjustments. The brown line represents positive correlation, the blue line represents negative correlation, and the thickness and thinness of the line represent strong and weak correlation, respectively

Metabolic network diagrams related to “Antioxidant”, “Energy Metabolism”, and “Lipid Metabolism” pathways were established to investigate the relationship between rumen microbiota, rumen metabolites, plasma metabolites, and host phenotype. Within the “Antioxidant” category, up-regulated *Lactobacillus_terrae* and *Pediococcus_pentosaceus,* along with down-regulated *Shinella*, *Acinetobacter junii*, and *Vibrio kanaloae* in the rumen were associated with changes in ruminal and plasma metabolites, ultimately exhibiting positive and negative correlations, respectively, with sperm motility (*P* < 0.05; Fig. 9A). Within the “Energy Metabolism” category, up-regulated *Romboutsia*, *Turicibacter*, *Clostridium *sensu stricto* 1*, NK4A214_group, *Bacteroides acidifaciens* along with down-regulated *Acidaminococcus_fermentans* and EMP-G18 in the rumen were associated with changes in ruminal and plasma metabolites, ultimately exhibiting positive and negative correlations, respectively, with sperm motility (*P* < 0.05; Fig. 9B). Within the “Lipid Metabolism” category, up-regulated *Akkermansia*, *Clostridium *sensu stricto* 1*, *Coriobacteriaceae* UCG-002, *Enterorhabdus*, *Porphyromonas*, *Paramuribaculum intestinale* along with down-regulated Hyphomicrobium and *Acidaminococcus fermentans* in the rumen were associated with changes in ruminal and plasma metabolites, thereby exhibiting positive and negative correlations, respectively, with sperm motility (*P* < 0.05; Fig. 9C).

## Discussion

This study demonstrates that dietary supplementation with NAC significantly enhances sperm motility and overall semen quality in Qianbei Ma goats. Notably, our multi-omics approach unravelled a novel mechanism by which NAC exerts its beneficial effects: by remodelling the rumen microbiota and its metabolic output, which systemically influences host reproductive function. These findings position the rumen microbiome as a crucial mediator in the interplay between nutrition and male fertility [33], offering a new perspective on dietary intervention strategies for improving reproductive performance [34].

The rumen and hindgut serve as crucial sites for nutrient digestion and absorption in ruminants [35]. Given that the fermentative capacity of the hindgut accounts for only approximately 14% of that of the rumen [21], this study primarily focused on the role of the ruminal microbiota. The rumen’s function is underpinned by its complex microbial ecosystem, and findings identify a NAC-modulated rumen microbial community that enhances sperm motility. Within this system, we observed enrichment of beneficial bacteria such as *Pediococcus_pentosaceus* [36] and *Lactobacillus_terrae* [37], and the suppression of potential harmful bacteria, including *Acinetobacter junii* [38] and *Vibrio kanaloae* [39], indicating a structurally and functionally significant shift in the rumen microbiota. These changes were not captured by alpha diversity metrics but were clearly evident in functional potential and metabolite profiles, suggesting that functional and metabolic alterations may serve as more sensitive indicators of microbial ecological shifts than broad taxonomic composition [40]. Supporting the concept of a gut–plasma metabolic axis [41], our analysis revealed a substantial continuity between the ruminal and systemic metabolomes, with shared pathways accounting for 33.78% and 55.56% of the rumen fluid and plasma metabolomes, respectively. These findings indicate that systemic metabolic profiles are substantially influenced by ruminal processes, further supporting the central role of the rumen as the primary site of nutrient metabolism in ruminants [22].

The functional enrichment of microbial pathways in “Antioxidant Metabolism”, “Energy Metabolism”, and “Lipid Metabolism” provides a plausible mechanistic link between the altered microbiota and the improved sperm phenotype [25]. Specifically, the enhancement of antioxidant-related pathways, such as glutathione [42], lipoic acid [43], ascorbate and aldarate [44] metabolism, is of paramount importance. The significant positive correlations between the enriched microbes (*Pediococcus_pentosaceus* and *Lactobacillus_terrae*), antioxidant metabolites (e.g., urolithin B [45] and benzoic acid [46]), and sperm motility parameters strongly suggest that NAC fosters a microbial environment capable of mitigating systemic oxidative stress [33]. This was confirmed by significant functional classification pathways in the metabolomes of rumen fluid (glutathione, ascorbate and aldarate, lipoic acid, taurine, hypotaurine metabolism, etc.), plasma (glutathione, ascorbate, and aldarate metabolism, as well as ubiquinone and other terpenoid quinone biosynthesis, etc.), and changes in metabolic in pathways. This is consistent with the observed improvements in sperm antioxidant enzyme activities (T-AOC, SOD, GSH-Px) and reduction in lipid peroxidation (MDA) in spermatozoa. We propose that NAC, by providing a reducible thiol group, creates a more reduced ruminal environment that selectively favours the growth of antioxidant metabolite-producing bacteria, thereby enhancing sperm quality through systemic antioxidant effects. Nevertheless, the sperm concentration in this study appears lower than that reported in some previous studies, which may be attributable to variation in breed/genetic background, season/photoperiod, and local management conditions.

Beyond its antioxidant properties, our results indicate that NAC supplementation significantly upregulates microbial energy metabolism pathways within the rumen, a phenomenon consistent with effects reported for other antioxidant supplements [47]. Specifically, NAC improves mitochondrial tricarboxylic acid (TCA) cycle metabolism by stimulating carbon flux through pyruvate dehydrogenase (PDH) [48], a mechanism that may be conserved in its enhancement of ruminal microbial energy harvesting and conversion. In the sequencing of rumen fluid microbiota, critical pathways including the citrate cycle (TCA cycle), oxidative phosphorylation, pyruvate metabolism, propanoate metabolism, butanoate metabolism, as well as valine, leucine, and isoleucine degradation were significantly enriched, suggesting that NAC, like other antioxidants, enhanced microbial energy harvesting and conversion [49, 50]. Specific bacteria, such as *Bacteroides acidifaciens* and *Romboutsia*, were notably associated with these pathways, which is consistent with their known roles in optimising host energy harvest [51, 52]. These metabolic changes were corroborated by alterations in both the rumen and plasma metabolomes. Specifically, ruminal metabolites associated with glycolysis/gluconeogenesis, TCA cycle, and amino acid degradation were elevated, while systemic changes were reflected in the plasma through pathways such as carbon metabolism, 2-oxocarboxylic acid metabolism, pantothenate and CoA biosynthesis, and thiamine metabolism. Key ruminal intermediates—including arginine, Gly-Asp, levulinic acid, and tryptophan—showed positive correlations with beneficial microbes such as *Bacteroides acidifaciens* [53] and *Romboutsia* [54]. These shifts extended systemically, as evidenced by elevated plasma levels of energy-related metabolites like N-methyl-L-asparagine and D-glutamine, which correlated positively with sperm motility parameters [55, 56]. The finding that intragastric administration of N-methyl-L-asparagine in mice significantly enhanced sperm motility provides direct evidence that microbial-derived energy metabolites are absorbed into circulation and functionally contribute to sperm kinetic performance, likely by enhancing ATP synthesis and mitochondrial energy production, similar to the effects of L-aspartic acid [57, 58]. However, given inherent interspecies differences between mice and goats, these mouse data should be interpreted as supportive rather than definitive for ruminants, and future work will prioritize goat-based in vitro and/or in vivo validation of the identified molecules to confirm their species-specific roles in regulating sperm function.

Concurrently, NAC supplementation markedly remodelled microbial lipid metabolism in the rumen. Functional analysis revealed significant enrichment of key lipid-related pathways—including biosynthesis of unsaturated fatty acids, glycerophospholipid metabolism, and steroid hormone biosynthesis—suggesting enhanced microbial capacity for lipid metabolism and modification under NAC supplementation [59, 60]. These functional shifts were corroborated by metabolomic changes in both the rumen and plasma. In ruminal fluid, we observed elevated activity in glycerophospholipid, ether lipid, and glycerolipid metabolic pathways. Correspondingly, systemic alterations were detected in the plasma, involving glycerophospholipid metabolism, sphingolipid metabolism and signalling, arachidonic acid metabolism, and linoleic acid metabolism. These changes were reflected in the rumen metabolome through increased levels of bioactive lipids and signalling molecules such as homononactinic acid [61], N-decanoyl-L-homoserine lactone [62], and several glycerophospholipids [63]. Notably, the systemic influence of microbiota-driven lipid remodelling was reflected in the plasma bile acid profile: sperm motility parameters correlated positively with taurine-conjugated bile acids, which are known to enhance sperm motility [64, 65], whereas a negative correlation was observed with glycine-conjugated species, associated with asthenozoospermia [66]. This indicates that NAC stimulates microbial production of essential structural and signalling lipids and modulates the composition of bioactive bile acids, which regulate systemic lipid homeostasis and cellular membrane function [67]. We propose that microbial-derived lipid metabolites—particularly those absorbed into circulation—support sperm motility by contributing to membrane fluidity and stability, while also acting as signalling molecules that facilitate sperm physiological function.

﻿An innovative aspect of the current study is the delineation of a potential “rumen–plasma–sperm” axis, adding a new layer of mechanistic understanding to the established concept of the gut–testis axis [68]. We identified specific microbial taxa whose abundance shifts were correlated with changes in both ruminal and plasma metabolites through functional connections, along with overlapping metabolic pathways between the rumen and plasma, ultimately linking to the sperm motility phenotype. This cascade suggests a coherent transfer chain whereby NAC-modulated microbial metabolites are absorbed into the systemic circulation and function as signalling molecules to distantly enhance sperm motility. Specifically, these metabolites systemically improve sperm's antioxidant status, energy supply, and lipid homeostasis, thereby elevating overall semen quality. This study highlights the rumen microbiome as a promising therapeutic target for improving animal reproduction and provides a foundational framework for future research. Future studies involving microbial transplantation or targeted probiotics could further solidify the causal role of these microbes in the observed benefits.

## Conclusion

In conclusion, dietary NAC supplementation significantly enhanced sperm motility in goats, with the optimal effect observed at 0.3%. NAC promoted the enrichment of beneficial bacteria, whose remodeling led to the functional enrichment of metabolic pathways related to antioxidant capacity, energy production, and lipid metabolism. These metabolic alterations ultimately enhanced sperm motility by improving sperm antioxidant status, energy supply, and lipid homeostasis. Collectively, our findings propose a novel “rumen–plasma–sperm” metabolic axis and highlight the promise of NAC-based dietary interventions for improving reproductive performance.

## Supplementary Information

Additional file 1: Table S1. Sperm motility of male Qianbei Ma goats in each group before feeding. Table S2. Body weight of male Qianbei Ma goats in each group before feeding. Table S3. Diet ingredient and nutritional levels. Table S4. Primer sequence information. Table S5. The relative abundances of ruminal KEGG pathways between Control and 0.3% NAC Qianbei Ma goats.

Additional file 2: Fig. S1. Effect of NAC on the sperm functional parameters of the Qianbei Ma goats. Fig. S2. Effect of NAC on the rumen microbiota of Qianbei Ma goats. Fig. S3. NAC affects metabolite changes in rumen fluid. Fig. S4. NAC affects metabolite changes in plasma.

## Data Availability

Sequence data that support the findings of this study have been deposited in the China National GeneBank DataBase (CNGBdb) with the primary accession code CNP0008314. These data can be accessed at the following link: https://doi.org/10.26036/CNP0008314.

## Abbreviations

ASV: Amplicon sequence variants
CAT: Catalase
DEG: Differentially expressed gene
FSH: Follicle-stimulating hormone
GnRH: Gonadotrophin-releasing hormone
GO: Gene ontology
GSH: Glutathione
GSH-Px: Glutathione peroxidase
GSSG: Oxidized glutathione
KEGG: Kyoto Encyclopedia of Genes and Genomes
LH: Luteinising hormone
MDA: Malondialdehyde
MMP: Mitochondrial membrane potential
NAC: N-acetylcysteine
SOD: Superoxide dismutase
T-AOC: Total antioxidant capacity
