# Supplementary material for: Dietary N-acetylcysteine enhances sperm motility by remodeling the rumen microbiome and its metabolic axis in goats
Source: J Anim Sci Biotechnol. 2026 Apr 20;17:73. doi: 10.1186/s40104-026-01390-2 (PMC13093934; doi:10.1186/s40104-026-01390-2)
Supplement: Supplementary file 2 — Additional file 2: Fig. S1. Effect of NAC on the sperm functional parameters of the Qianbei Ma goats. Fig. S2. Effect of NAC on the rumen microbiota of Qianbei Ma goats. Fig. S3. NAC affects metabolite changes in rumen fluid. Fig. S4. NAC affects metabolite changes in plasma. [file 40104_2026_1390_MOESM2_ESM.docx]

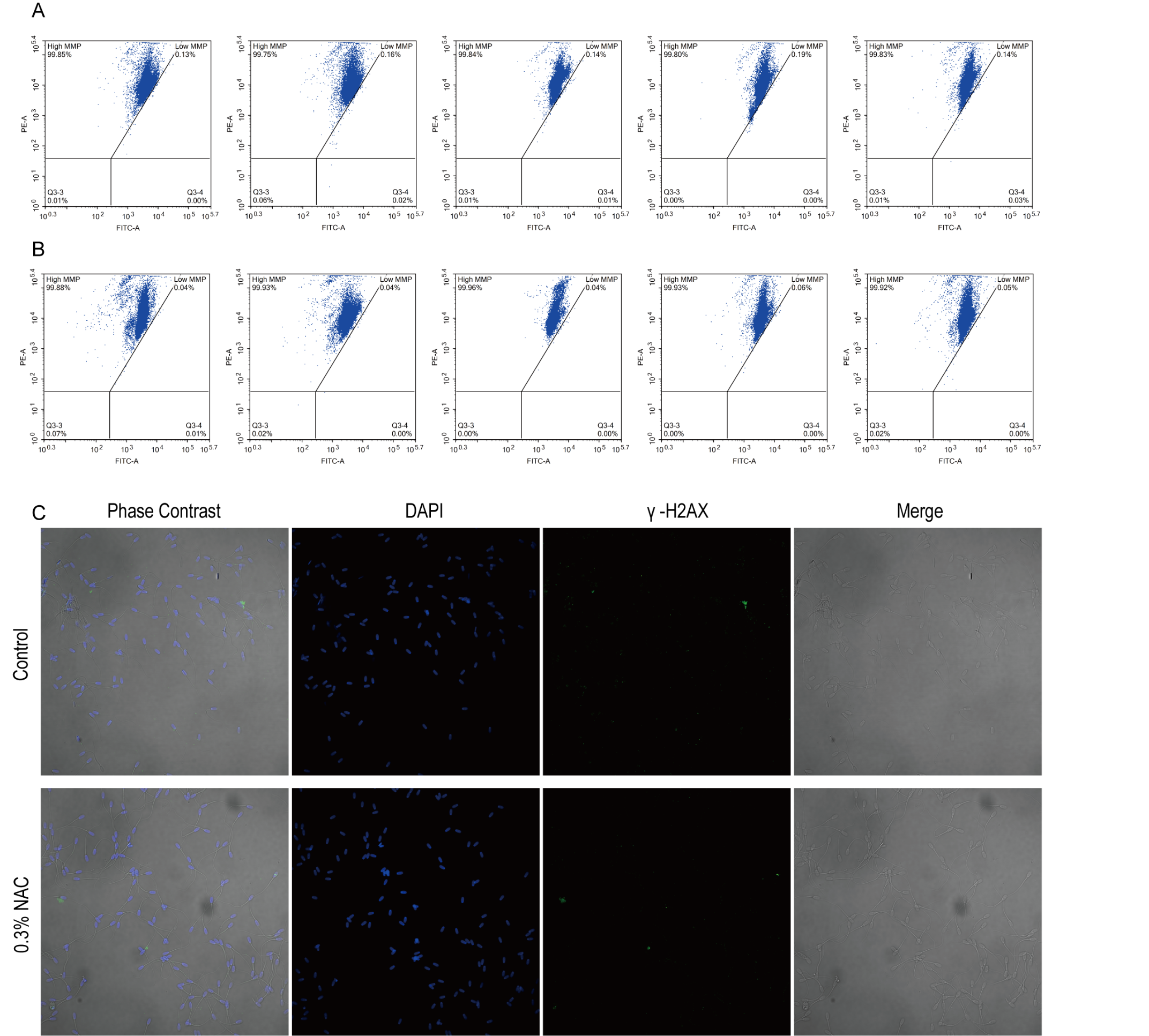


**Fig. S1.** Effect of NAC on the sperm functional parameters of the *Qianbei Ma* goats. **A** Sperm populations with high and low membrane potentials identified using flow cytometry in the control group. **B** Sperm populations with high and low membrane potentials identified using flow cytometry in the 0.3% NAC group. **C** DNA damage detected by γ-H2AX immunofluorescence assay.


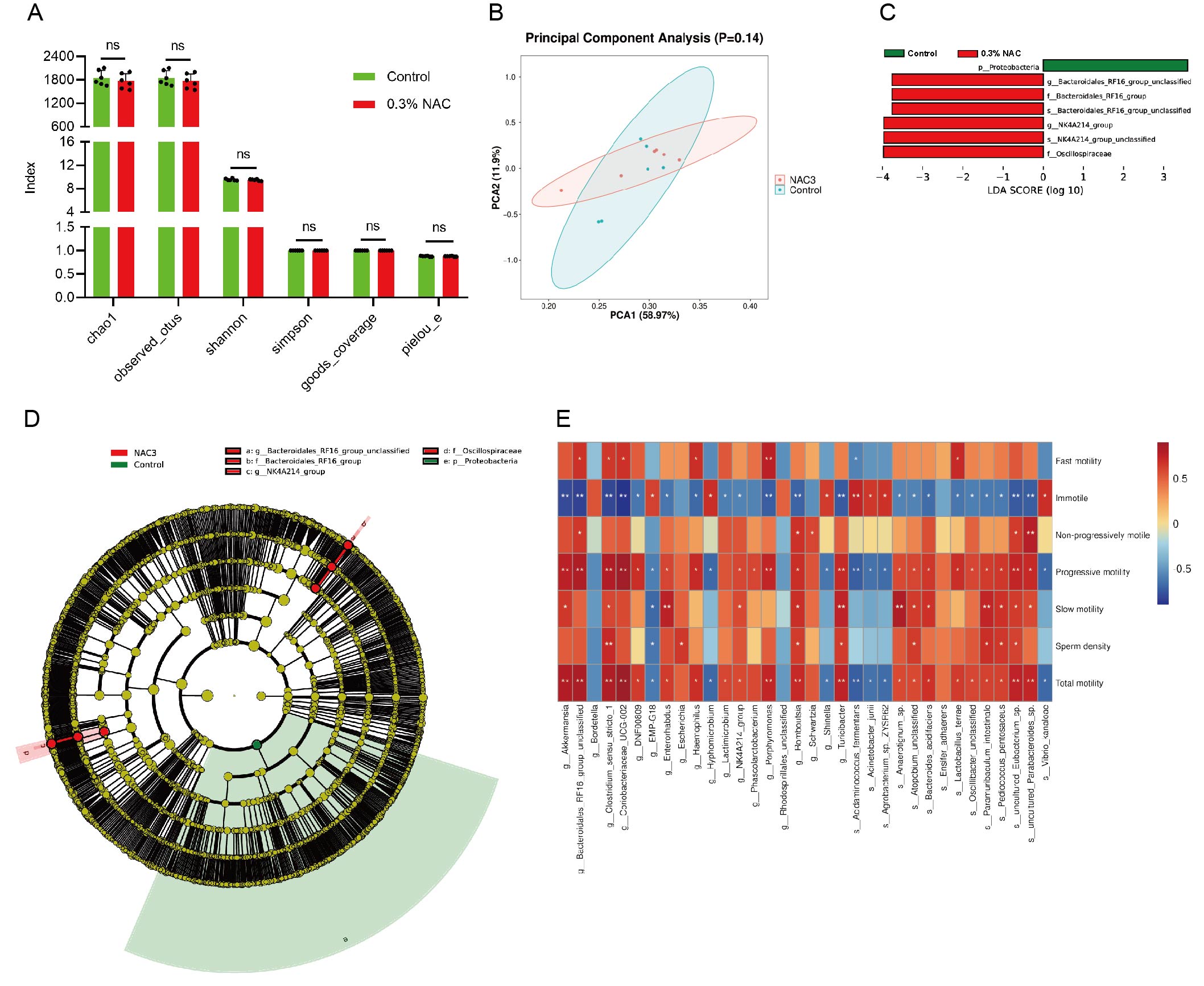


**Fig. S2.** Effect of NAC on the rumen microbiota of *Qianbei Ma* goats. A Alpha diversity. **B** PCA of β diversity. **C** Evolutionary cladogram. **D** Influence distribution histogram. **E** Spearman correlation coefficient between all rumen microbiota and host phenotype


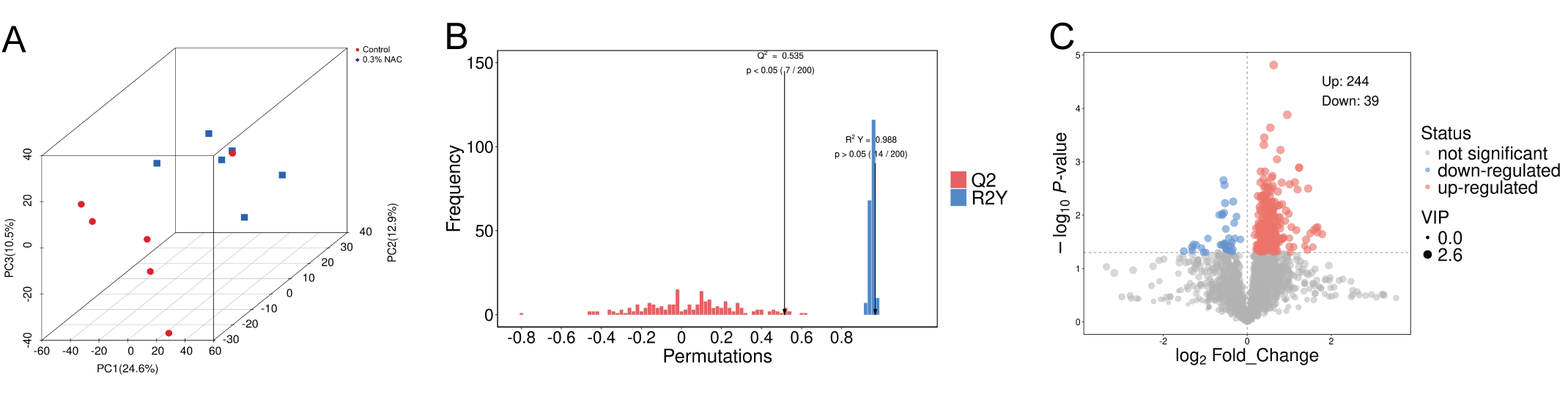


**Fig. S3.** NAC affects metabolite changes in rumen fluid. **A** Unsupervised PCA was conducted to evaluate differences among different groups. **B** OPLS-DA permutation histogram. **C** Volcano plot of differentially abundant metabolites.


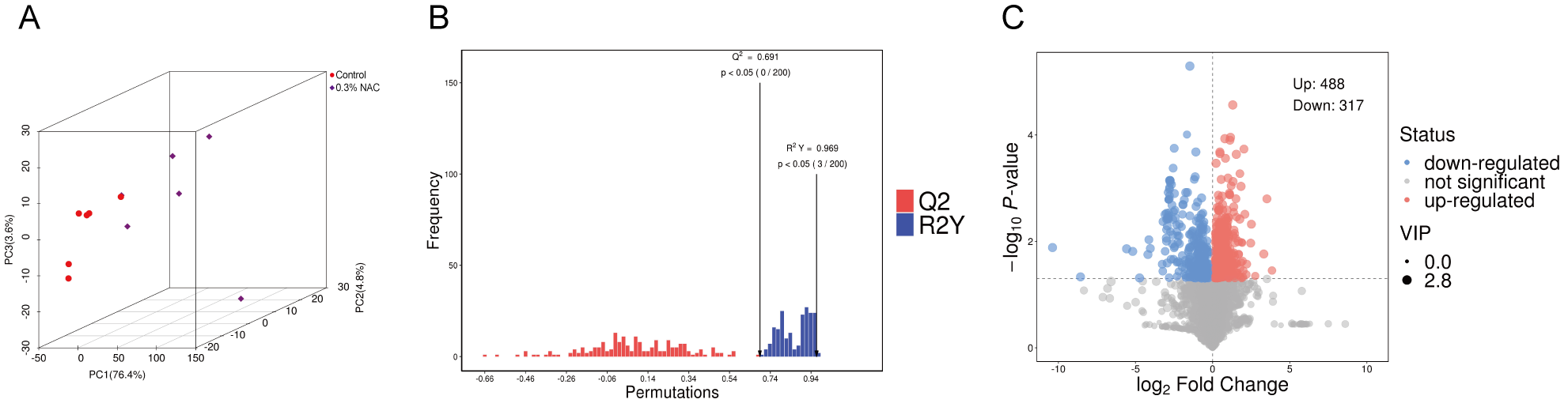


**Fig. S4.** NAC affects metabolite changes in plasma. **A** Unsupervised PCA was conducted to evaluate differences among different groups. **B** OPLS-DA permutation histogram. **C** Volcano plot of differentially abundant metabolites.
